# Menu provision in a young offenders institution, comparison with dietary guidelines, and previous menu allocation: a cross-sectional nutritional analysis

**DOI:** 10.1017/jns.2024.62

**Published:** 2024-10-08

**Authors:** Matthew Poulter, Shelly Coe, Catherine Anna-Marie Graham, Bethan Leach, Jonathan Tammam

**Affiliations:** 1 Centre for Nutrition and Health, Faculty of Health & Life Sciences, Oxford Brookes University, Oxford, UK; 2 Center for Interdisciplinary Research (CEFIR), Cereneo Foundation, Vitznau, Switzerland; 3 Lake Lucerne Institute, Vitznau, Switzerland; 4 Practice Plus Group, Berkshire, UK

**Keywords:** Incarceration, Nutritional analysis, Nutrition, Prisoners, Public health, Sugar, Vitamin D, Vitamin D deficiency, Vulnerable population, Young offenders institution

## Abstract

*Objective:* This study aimed to assess and comparatively analyse two menus from a Young Offenders Institution (YOI). One menu from 2019, and one from 2022, with the objective of identifying any improvements in meeting dietary guidelines. *Design:* Cross-sectional and comparative analysis. *Setting:* United Kingdom, a YOI in Northern England. *Participants:* YOI Menus. *Results:* Analysis of 30 dietary components identified that 25 exceeded the dietary guidelines (P < 0.05) for the 2022 menu, with five failing to meet the guidelines (P < 0.05). When compared to the 2019 menu, the 2022 menu showed improvements in saturated fat, sodium, and vitamin D. Despite the improvement, vitamin D levels remained below dietary guidelines (P < 0.01). Salt and energy content were reduced in the 2022 menu (P < 0.05); however, they were still above the dietary guidelines (P < 0.01). Free sugars were significantly above dietary guidelines for both menus, with no significant change between the 2019 and 2022 menu (P = 0.12). *Conclusion:* The 2022 menu has demonstrated progress in alignment with meeting dietary guidelines, particularly in reducing calories, fat, saturated fat, salt, sodium, and chloride, as well as increasing vitamin D. Despite improvements, calories, free sugars, salt, saturated fat, sodium, and chloride are still exceeding dietary guidelines, posing as potential health risks.

## Introduction

A healthy diet is widely known for its importance to overall human health and development. Nutrient deficiencies are linked to a higher risk of poor physical and mental health,^(1,2)^ reduced cognitive function, lower educational outcomes, and diminished productivity.^(3,4)^ Specific population groups face a higher risk of poor nutrition, including populations in poverty, experiencing food insecurity, and those lacking the ability to control their own diet.^(5–7)^ Prisoners are an example of such a vulnerable population.

Prisoners’ loss of liberty results in a reduction of autonomy over aspects of their lives, with one key aspect being their diet. Although prisoners are presented with menu choices, for example, five options at lunch and dinner, they remain restricted to a diet institutionally provided for them.^(8)^

This food provision is constrained by the restricted budget which the catering manager must work with, estimated to be approximately only £2 per prisoner per day, though this can be less in some cases.^(8)^ For comparison, National Health Service (NHS) hospitals, on average, spent £4.56 per patient meal in 2018–19, though as with prisons, this budget can vary.^(9)^ While on the surface, hospitals also appear to suffer from a low budget, it is worth noting that hospital patients have a much shorter stay, with 5.2 months in 2020–21, compared with the average custodial sentence of 21.4 months in 2022.^(10,11)^

In 2022, it was estimated in England and Wales that just over 13,000 children aged between 10 and 17 received cautions or sentences from the courts.^(12)^ The majority of those sentenced will serve this within a Young Offenders Institution (YOI), designed for individuals aged 15–21. As of March 2022, the average monthly population in these institutions was 454.^(12,13)^

Prisoner health is compromised, with them suffering a disproportionately higher burden of physical and mental health conditions compared to the general population. Up to 90% across the prison estates are estimated to have at least one undiagnosed mental health condition.^(14)^ For those in youth justice, practitioners have reported that just over 70% of young offenders present with mental health concerns and that they suffer from a higher burden than adolescents in the general population.^(15,16)^ In many cases, this is attributable to traumatic life experiences, such as witnessing family violence, abuse, substance misuse issues, and neglect.^(16)^

A high cost is associated with youth justice, estimated at £300 million across the youth estates in 2019, encompassing YOIs, secure children’s homes, and training settings.^(17)^ For YOIs, the expenditure was estimated at around £76,000 per young offender per year.^(18)^ This represents a substantial cost to taxpayers, though it is a cost which could be reduced through improved diet quality and living conditions.^(14)^

Nutrition is crucial for one’s overall health, and institutions housing vulnerable young offenders must ensure that they meet nutritional standards. Therefore, it is critical to identify the nutritional profile of the food offered to prisoners, ensuring they have access to adequate nutrition. This is not only beneficial for their health but also for their future in society. His Majesty’s Prison and Probation Service (HMPPS) is currently developing new menus for male and female adult prisons, as well as Young Offenders Institutions (YOIs). The aim of these new menus is to ensure that dietary options enable prisoners to meet the Government Dietary Recommendations (GDRs).^(19)^

The objective of this study was to assess the nutritional content of a current and previous menu, developed by HMPPS for a male YOI in Northern England. Specific aims include comparing both menus’ nutritional content to (i) the UK GDRs, an update on the formerly used dietary reference values (DRVs),^(19)^ (ii) Tolerable Upper Intake Level (ULs), which represent the maximum daily intake of a nutrient which is unlikely to pose a negative risk to one’s health, as defined by the European Food Safety Authority (EFSA),^(20)^ (iii) and a comparison in nutritional content between the 2019 and 2022 menus.

## Materials and methods

Two menus were provided by HMPPS, both of which were used in a YOI in Northern England, one from 2019 and one from 2022. While YOIs across England are provided with food from the same supplier, and most follow a similar menu format, we cannot state that this menu analysed will be fully representative of other YOIs in England. This is due to the freedom catering managers have in what main meals they prepare, in addition to meeting the demands of prisoners with special dietary needs, i.e. halal, vegetarian etc, which can vary across the prison estates. However, typically what is provided for breakfast, snack, and dessert options will remain similar across prisons, with only lunch and dinner options open to some variation.

Each menu consisted of 28 days over a four-week cycle. The nutrient profile of the menus was compared to the GDRs, with additional comparisons to the UL by EFSA due to an absence of guidelines provided by the UK Government. Regarding the UL, there is only data for n = 10 dietary components, in the cases where there is no UL provided, this is usually due to limited data to derive a UL for the nutrient in question.^(20)^ This analysis used the UL values provided for the age groups of 15–17 and 18+, as the YOI houses prisoners aged between 15 and 18 years.

### Statistical analyses

Recipes were provided by the catering supplier and entered into the nutrition software Nutritics (v5.80, Dublin, Ireland) to calculate the nutritional content of each recipe for an individual portion.^(21)^ Recipe data were organised in Microsoft Excel and exported to IBM SPSS Statistics (v29.0, New York, NY, USA) for Macintosh.^(22)^ The final analysis was a comparison of the nutrient profiles between the new (2022) and old (2019) menu to identify changes in nutritional content between the two. Data were assessed for normality using the Shapiro–Wilk test. When comparing macro and micronutrient menu data to the GDRs, and ULs, the One-Sample *T*-Test was used for normally distributed data, and the Wilcoxon One-Sample Signed-Rank test for non-normal data. When comparing the old (2019) and new (2022) menu an Independent Samples *t*-Test was conducted for normally distributed data, and The Mann–Whitney *U* test for non-normally distributed data. Data were considered significant at P-value (P < 0.05).

## Results

### Comparison of new (2022) menu provision to GDRs

Out of the n = 30 dietary components tested, all results were found to differ from their GDR target (P < 0.05), with n = 24 exceeding their respective GDR and n = 6 failing to meet their target. See Table 1 for a full list of dietary components, and results.

**Table 1.** Comparative analysis of macro and micronutrient composition of the 2022 28-day menu provision and UK Government dietary recommendations for young males aged 15–18 years old

Dietary component	Mean	SD	GDR target^*^	Mean difference	Sig.
Energy (kcal)	2696	97	2500	196	<0.01^a^
Macronutrients					
Carbohydrates (g)	388	19	333	55	<0.01^a^
Free sugars (g)	63^‡^	8	33^†^	30	<0.01^b^
Fat (g)	88	7	97^†^	–9	<0.01^a^
Monounsaturated fat (g)	27	3	36	–9	<0.01^a^
Polyunsaturated fat (g)	12	2	18	–6	<0.01^a^
Saturated fat (g)	33	3	31^†^	2	<0.01^a^
Fibre (g)	39	4	30	9	<0.01^a^
Protein (g)	84.8	5.4	55.2	29.6	<0.01^a^
Salt (g)	8^‡^	1	6^†^	2	<0.01^b^
Vitamins					
Folates (μg)	390	25	200	190	<0.01^a^
Niacin (mg)	39.1	1.9	16.5	22.6	<0.01^a^
Riboflavin (mg)	2.2	0.2	1.3	0.9	<0.01^a^
Thiamine (mg)	3	0.4	1	2	<0.01^a^
Vitamin A (μg)	981^‡^	218	700	281	<0.01^b^
Vitamin B12 (μg)	4.9	0.5	1.5	3.4	<0.01^a^
Vitamin B6 (mg)	2.8	0.1	1.5	1.3	<0.01^a^
Vitamin C (mg)	129	21	40	89	<0.01^a^
Vitamin D (μg)	8	0.8	10	–2	<0.01^a^
Minerals					
Calcium (mg)	1118	103	1000	118	<0.01^a^
Chloride (mg)	4960^‡^	79	2500	2460	<0.01^b^
Copper (mg)	2.2	0.2	1	1.2	<0.01^a^
Iodine (μg)	131	17	140	–9	<0.01^a^
Iron (mg)	21	2	11	10	<0.01^a^
Magnesium (mg)	371	25	300	71	<0.01^a^
Phosphorus (mg)	1494	84	775	719	<0.01^a^
Potassium (mg)	4260	338	3500	760	<0.01^a^
Selenium (μg)	47	6	70	–23	<0.01^a^
Sodium (mg)	2986^‡^	452	2400	586	<0.01^b^
Zinc (mg)	15.3	0.6	9.5	5.8	<0.01^a^

SD, standard deviation; GDR, Government Dietary Recommendations; kcal, calories; g, grams; μg, micrograms; mg, milligrams.

a One-Sample *t* Test.

b One-Sample Wilcoxon Signed Rank Test.

* GDR target is based on the values for males aged between 15 and 18.

† Indicates that the GDR is the maximum allowance for this dietary component.

‡ Median.

### Comparison of new (2022) menu to upper intake limit

UL values are provided for only n = 10 dietary components. N = 9 were found to be below their UL value (P < 0.01), while only magnesium was found to be exceeding its UL figure (P < 0.01) (Table 2).

**Table 2.** Comparison of macro and micronutrient content in the 2022 28-day menu with EFSA tolerable upper intake Levels for young males aged 15–18 years old

	Mean	UL (ages 15–17)	Mean Difference	UL (age 18)	Mean Difference	Within UL	Sig.
Minerals							
Calcium (mg/day)	1118	N.D.	N.D.	2500	N.D.	Yes	<0.01^a,c^
Copper (mg/day)	2.2	4	–1.8	5	–2.8	Yes	<0.01^a,c^
Iodine (μg/day)	131	500	–369	600	–469	Yes	<0.01^a,c^
Folates (μg/day)	390	800	–410	1000	–610	Yes	<0.01^a,c^
Magnesium (mg/day)	371	250^d^	121	250^d^	121	No	<0.01^a,c^
Selenium (μg/day)	47	250	–203	300	–253	Yes	<0.01^a,c^
Zinc (mg/day)	15	22	–7	25	–10	Yes	<0.01^a,c^
Vitamins							
Vitamin A (μg/day)	981^‡^	2600	–1619	3000	–2019	Yes	<0.01^b,c^
Vitamin B6 (mg/day)	3	20	–17	25	–22	Yes	<0.01^a,c^
Vitamin D (μg/day)	8	100	–92	100	–92	Yes	<0.01^a,c^

UL, tolerable upper intake level; mg, milligrams; N.D., no data; μg, micrograms; EFSA, European Food Safety Authority.

a One-Sample *t* Test.

b One-Sample Wilcoxon Signed Rank Test.

c Result is significant for both UL (15–17), and UL (18).

d Does not include magnesium naturally present in food or beverages.

‡ Median.

### Comparison of old (2019) menu provision to GDRs

Out of the n = 30 dietary components tested, n = 29 were found to be significantly different, with n = 25 found to be exceeding their respective GDR target, and n = 4 significantly below (P < 0.05) (Table 3).

**Table 3.** Comparative analysis of macro and micronutrient composition of the 2019 28-day menu provision and UK Government dietary recommendations for young males aged 15–18 years old

Dietary component	Mean	SD	GDR target^*^	Mean difference	Sig.
Energy (kcal)	2903^‡^	240	2500	403	<0.01^b^
Macronutrients					
Carbohydrates (g)	406^‡^	37	333	73	<0.01^b^
Free sugars (g)	61^‡^	14	33^†^	28	<0.01^b^
Fat (g)	102	11	97^†^	5	<0.01^a^
Monounsaturated fat (g)	33	4	36	–3	<0.01^a^
Polyunsaturated fat (g)	12^‡^	2	18	–6	<0.01^b^
Saturated fat (g)	38	6	31^†^	7	<0.01^a^
Fibre (g)	34	3	30	4	<0.01^a^
Protein (g)	83.1^‡^	6.4	55.2	27.9	<0.01^b^
Salt (g)	10	2	6^†^	4	<0.01^a^
Vitamins					
Folates (μg)	321	28	200	121	<0.01^a^
Niacin (mg)	39.8^‡^	2.9	16.5	23.3	<0.01^b^
Riboflavin (mg)	2.1	0.3	1.3	0.8	<0.01^a^
Thiamine (mg)	2.4	0.3	1	1.4	<0.01^a^
Vitamin A (μg)	937^‡^	355	700	237	<0.01^b^
Vitamin B12 (μg)	3.7^‡^	0.9	1.5	2.2	<0.01^b^
Vitamin B6 (mg)	2.3	0.2	1.5	0.8	<0.01^a^
Vitamin C (mg)	103^‡^	18	40	63	<0.01^b^
Vitamin D (μg)	2^‡^	1	10	–8	<0.01^b^
Minerals					
Calcium (mg)	1252^‡^	202	1000	252	<0.01^b^
Chloride (mg)	6146	1080	2500	3646	<0.01^a^
Copper (mg)	1.5	0.2	1	0.5	<0.01^a^
Iodine (μg)	147^‡^	33	140	7	<0.05^b^
Iron (mg)	15	2	11	4	<0.01^a^
Magnesium (mg)	327	30	300	27	<0.01^a^
Phosphorus (mg)	1467^‡^	161	775	692	<0.01^b^
Potassium (mg)	3934	270	3500	434	<0.01^a^
Selenium (μg)	46	6	70	–24	<0.01^a^
Sodium (mg)	3849	597	2400	1449	<0.01^a^
Zinc (mg)	9.3	0.8	9.5	–0.2	0.22^a^

SD, standard deviation; GDR, Government Dietary Recommendations; kcal, calories; g, grams; μg, micrograms; mg, milligrams.

a One-Sample *t* Test.

b One-Sample Wilcoxon Signed Rank Test.

* GDR target is based on the values for males aged between 15 and 18.

† Indicates the GDR is the maximum allowance for this dietary component.

‡ Median.

### Comparison of old (2019) menu to upper intake limit

Of the 10 UL values provided, n = 9 dietary components were found to be below their UL value (P < 0.01) (Table 4). While only magnesium was found to be exceeding its UL figure (P < 0.01).

**Table 4.** Comparison of macro and micronutrient content in the 2019 28-day menu with EFSA tolerable upper intake Levels for young males aged 15–18 years old

	Mean	UL (ages 15–17)	Mean difference	UL (age 18)	Mean difference	Within UL	Sig.
Minerals							
Calcium (mg/day)	1202^‡^	N.D.	N.D.	2500	–1298	Yes	<0.01^b,c^
Copper (mg/day)	1.5	4	–2.5	5	–3.5	Yes	<0.01^a,c^
Iodine (μg/day)	147^‡^	500	–353	600	–453	Yes	<0.01^b,c^
Folates (μg/day)	321	800	–479	1000	–679	Yes	<0.01^a,c^
Magnesium (mg/day)	327	250^d^	77	250^d^	77	No	<0.01^a,c^
Selenium (μg/day)	46	250	–204	300	–254	Yes	<0.01^a,c^
Zinc (mg/day)	9	22	–13	25	–16	Yes	<0.01^a,c^
Vitamins							
Vitamin A (μg/day)	937^‡^	2600	–1663	3000	–2063	Yes	<0.01^b,c^
Vitamin B6 (mg/day)	2	20	–18	25	–23	Yes	<0.01^a,c^
Vitamin D (μg/day)	2^‡^	100	–98	100	–98	Yes	<0.01^b,c^

UL, tolerable upper intake level; mg, milligrams; N.D., no data; μg, micrograms; EFSA, European Food Safety Authority.

a One-Sample *t* Test.

b One-Sample Wilcoxon Signed Rank Test.

c Result is significant for both UL (15–17), and UL (18).

d Does not include magnesium naturally present in food or beverages.

‡ Median.

### Comparison of new (2022) and old (2019) menu

**Fig. 1.**
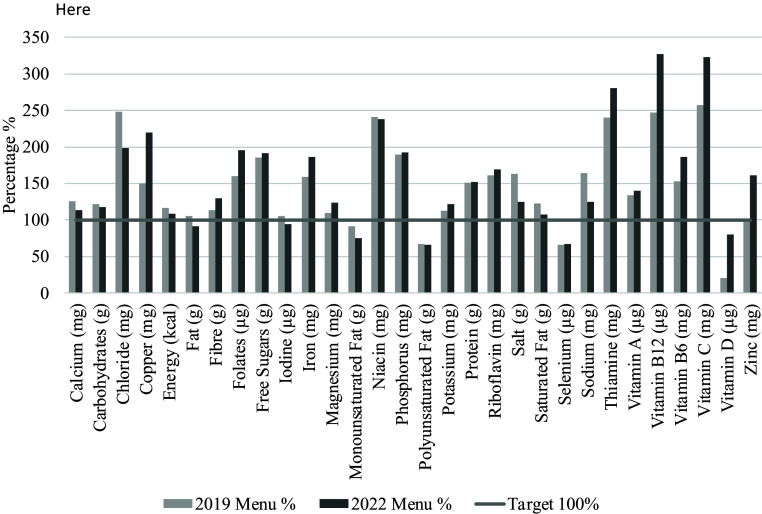
2022 and 2019 menu dietary components as a percentage, identifying those which have met their GDR target.

Regarding the results of macro and micronutrients, there were n = 22 significant differences between the two menus (P < 0.05). Of these, n = 13 was higher in the new menu, while n = 9 was lower (Table 5; Fig. 1). Of those which were higher in the new menu, these included copper, fibre, folates, iron, magnesium, potassium, riboflavin, saturated fat, zinc, vitamin B12, B6, C, and D (P < 0.05). The n = 9 which was lower in the new menu included calcium, chloride, energy, fat, iodine, monounsaturated fat, salt, sodium, and thiamine (P < 0.05).

**Table 5.** Comparative analysis of the dietary components from the 2019 and 2022 menus for male young offenders aged 15–18 years old

	2019 Menu		2022 Menu				
Dietary component	Mean	SD	Mean	SD	Mean diff.	95% CI	Sig.
Energy (kcal)	2903^*^	240	2705^*^	97	–198	–239, –104	<0.05^d^
Macronutrients							
Carbohydrates (g)	405^*^	37	390^*^	19	–15	–24, 0.8	0.06^d^
Free sugars (g)	61^*^	14	63^*^	8	2	–2, 10	0.12^d^
Fat (g)	102	11	88	7	–14	–19, –9	<0.01^a,b^
Monounsaturated fat (g)	33	4	27	3	–6	–8, –4	<0.01^a,b^
Polyunsaturated fat (g)	12.1^*^	1.6	11.9^*^	1.8	–0.2	–1, 0.9	0.96^d^
Saturated fat (g)	38	5.7	33.4	3.3	4.6	–7, –2	<0.01^a,c^
Fibre (g)	34	3	39	4	5	2, 6	<0.01^a,b^
Protein (g)	83.1^*^	6.4	83.9^*^	5.4	0.8	–0.5, 5.6	0.12^d^
Salt (g)	9.8^*^	1.5	7.5^*^	1.1	–2.3	–3, –1	<0.01^d^
Vitamins							
Folates (μg)	321	28	390	25	69	55, 83	<0.01^a,b^
Niacin (mg)	39.8^*^	3	39.2^*^	1.9	–0.6	–1.7, 0.8	0.51^d^
Riboflavin (mg)	2.1	0.3	2.2	0.2	0.1	0.02, 0.3	<0.05^a,c^
Thiamine (mg)	2.4	0.3	2.8	0.4	–0.4	0.2, 0.6	<0.01^a,b^
Vitamin A (μg)	936.5^*^	355.2	980.8^*^	216.7	44.3	–124, 101	0.90^d^
Vitamin B12 (μg)	3.7^*^	0.9	4.9^*^	0.5	1.2	0.7, 1.7	<0.01^d^
Vitamin B6 (mg)	2.3	0.2	2.8	0.2	0.5	0.5, 0.7	<0.01^a,b^
Vitamin C (mg)	103^*^	18	129^*^	21	26	13, 36	<0.01^d^
Vitamin D (μg)	2^*^	1	8^*^	1	6	6, 7	<0.01^d^
Minerals							
Calcium (mg)	1252^*^	202	1134^*^	103	–118	–186, –34	<0.05^d^
Chloride (mg)	6203^*^	1080	4960^*^	793	–1243	–1604, –443	<0.01^d^
Copper (mg)	1.5	0.2	2.2	0.2	0.7	0.6, 0.8	<0.01^a,b^
Iodine (μg)	147^*^	33	132^*^	17	–15	–41, –10	<0.01^d^
Iron (mg)	18	2	21	2	3	2, 4	<0.01^a,b^
Magnesium (mg)	327	30	371	25	45	30, 60	<0.01^a,b^
Phosphorus (mg)	1467.4^*^	161	1489.5^*^	83.5	22.1	–35, 99	0.39^d^
Potassium (mg)	3934.3	270.4	4259.5	338.4	325	161, 489	<0.01^a,b^
Selenium (μg)	46.1	6.1	47.1	6.4	–1	–2, 4	0.56^a,c^
Sodium (mg)	3937.1^*^	597.4	2986.2^*^	52.1	–950.9	–1107, –544	<0.01^d^
Zinc (mg)	9.3^*^	0.8	15.3^*^	0.6	6	5.6, 6.4	<0.01^a,b^

SD, standard deviation; CI, confidence intervals; kcal, calories; g, grams; μg, micrograms; mg, milligrams.

a Independent Samples *T* Test.

b Equal variances assumed.

c Equal variances not assumed.

d Independent-Samples Mann–Whitney *U* Test.

* Median value.

## Discussion

This study aimed to profile the menu offered to young male offenders in an English YOI and to identify changes between the new and old menus. Overall, the results indicated a positive improvement in the content of the 2022 menu, although there are still areas for improvement to note.

### 2022 menu and GDRs

In the 2022 menu, numerous instances were identified where dietary components exceeded the GDRs. These components included calcium, carbohydrates, chloride, copper, energy, fibre, folates, free sugars, iron, magnesium, niacin, phosphorus, potassium, protein, riboflavin, salt, saturated fat, sodium, thiamine, and vitamins A, B12, B6, C, and zinc. Most of these exceedances do not raise concerns, as further demonstrated by a comparison to the ULs, where for calcium, copper, folates, magnesium, vitamins A, B6, and zinc, these were below their respective ULs. Only magnesium exceeds both limits, however, the UL for magnesium applies to magnesium compounds found in nutritional supplements or added to food, not the naturally occurring magnesium in foods.^(23)^ Therefore, in this analysis, which only considered nutrients naturally present in foods, magnesium exceeding its UL limit is not of concern. It is worth noting that for 20 out of the 30 dietary components analysed, no ULs are established by the EFSA. The absence of UL values can be attributed to insufficient evidence, or the available evidence suggests that consuming these components at higher quantities than their respective GDR targets does not pose a major health risk.^(20,24)^

Carbohydrates, chloride, energy, fibre, free sugars, niacin, phosphorous, potassium, protein, riboflavin, salt, saturated fat, sodium, thiamine, vitamin B12, and C significantly exceeded their GDRs without an associated UL. Among these, saturated fat, free sugars, sodium, and salt standout as concerns as the GDRs state these should not be consumed above their targets. While the average quantities for saturated fat and salt exceed their respective GDR values by only 2 g, sodium and free sugars present a more significant concern, with free sugars nearly doubling the recommended 33g at 66g. These findings highlight a potential concern due to the associated health risks of a diet high in fat, sugar, and salt. High salt and sodium intake in adults can lead to hypertension and coronary heart disease, with evidence further suggesting similar risks for adolescents.^(25–29)^

With salt exceeding the GDR, it is then unsurprising that sodium is also exceeding its respective GDR. The health risks associated with high salt are similar to that of high sodium in the diet, including increasing risk of hypertension, chronic kidney disease, cardiovascular disease and potentially osteoporosis.^(30)^ Though these health risks are primarily evidenced in adults, a high salt diet from a young age can contribute to these potential health risks over time. The issues of high sodium in prisoners have been previously identified and reported as an area of concern. For example, Chrisostomou *et al.* (2019) investigated UK prisoner food choice, where choices led to sodium intake being significantly above the recommended intake (mean = 3056 mg, SD = 345, 95% CI = 2899–3214, P < 0.01).^(31)^ A more recent study by Johnson *et al.* (2022) looked at the nutrition content of menus in Canadian penitentiaries. Here sodium was also highlighted as exceeding the Canadian dietary reference intakes (DRIs) (1500 mg; mean = 3404.2), and this figure would also be exceeding UK recommendations.^(32)^

However, we did find that the menu was offering less salt and sodium than the intake of males (19–64y) based on the latest results from the National Diet and Nutrition Survey (NDNS). The estimated salt intake from the NDNS was reported as 9.2 g, and the sodium intake of 3256 mg.^(33)^ Though the results of the NDNS were for male adults, and this menu analysis is for adolescents, it was worth noting the general population intake to put the menu results into the context of the wider population. While no UL is provided by the EFSA for sodium, they do highlight evidence indicating sodium’s potential role in hypertension, putting one at risk of renal and cardiovascular issues.^(34)^ Johnson *et al.* (2022) noted that achieving a diet with sodium below the recommendations can be found unpalatable for many, with added salt found to improve the sensory properties of most foods for human consumption at a low cost.^(32,35)^ This becomes a difficult trade-off between providing a diet for prisoners which is palatable while minimising potential health risks from dietary components like sodium. However, the overall recommendation would be to reduce sodium or use low-sodium food alternatives.

Along with sodium, the high levels of free sugar (mean = 63 g, GDR = 33 g) almost double the maximum GDR allowance, pose potential health risks for young offenders due to increasing risk of weight gain, leading to obesity.^(36,37)^ In adult life, this can pose further risks for coronary heart disease and type-2 diabetes.^(38)^ Food items included in menus leading to this risk include sugar packets, and dessert items such as sponge cake, doughnuts, and biscuits. However, the problems with free sugars may be far worse, as we are only considering menu items. Prisoners also have the option to purchase their own foods from a prison shop (known in prison as the ‘canteen’) where, for example, Morley *et al.* (2019) demonstrated that prison shop food contained 12 times the amount of foods containing high levels of sugar, fat and salt based off the NHS Eatwell Guide reccomendations.^(39)^ Although shop purchases were not assessed in the current study, it can be assumed based on general prison food choice studies, that this will contribute negatively to total free sugar consumption. Future research should endeavour to assess all possible dietary provisions in prison settings, including dietary intake from the menu provided, and any shop purchases made. This will allow for the identification of food options available to prisoners which are contributing the most to the intake of free sugars, salt, sodium, and saturated fat, and in turn revaluation of these food items inclusion in prisons.

While the menu content identifies that energy intake was exceeding the GDR, it was only exceeded by 196 kcal. Any future changes to the menu would need to be mindful of this, as altering food options may reduce the average calories on offer. For example, the breakfast pack provided contained items such as cereal, sugar packets, tea, coffee, whitener, and hot chocolate. This was found to be a major cause for the high level of free sugars. When adjusting the breakfast pack and removing most of the high-sugar items, the menu almost met the GDR for free sugar with 34 g. However, this led to the new issue of the menu being below the GDR for energy intake with 2395 kcal. This highlights both the concern that many calories were coming from high-sugar items, as well as that any further changes to the menu would need to be mindful of a knock-on effect on the content of other dietary components.

Though much of this discussion has revolved around what is exceeding the GDRs, consideration must also be given to those below. In particular, we found monounsaturated fat, polyunsaturated fat, iodine, selenium, and vitamin D all below targets. There is a lower reference nutrient intake for selenium, which indicates that for males 18 + 40 μg per day will suffice, and the mean selenium in the menu was 47 μg.^(40)^ Therefore, selenium is not a huge concern in this menu, though improvements could be made. A broad recommendation would be to increase the menu in food items such as tofu and various fishes such as salmon, mackerel tuna, and herring, which would increase the levels of selenium, iodine, vitamin D, polyunsaturated and monounsaturated fat. Replacing red meat on the menu with options such as tofu and fish could decrease the total saturated fat content of the menu. However, given the budget in prisons, these options may not be financially feasible, but we would recommend further investigation of what foods are available by the suppliers to the prisons, to keep dietary choices in closer alignment with the GDRs.

### 2019 to 2022 menu comparison

Looking at the 2019 and 2022 menu comparison, although the 2022 menu analysis identifies areas for further improvement, overall, there were positive changes highlighting the efforts made to improve the nutrition on offer.

For example, fat content exceeded the GDR in 2019, and this has now been corrected in the 2022 menu with fat significantly below the GDR. The 2019 menu contained more use of beef in recipes, with eight beef-containing recipes, compared with the 2022 menu which reduced the total number down to just three. This would have had a major impact on the total fat content of each menu, and possibly a cause for the reduction of total fat, saturated fat, and monounsaturated fat. This also allows the new menu to be more aligned with government recommendations regarding reducing the consumption of red meat.^(41)^ By providing fewer options containing beef, the menu has improved upon this.

Many of the recipes containing beef were also high in carbohydrates, with recipes such as beef baguette, and lasagne, which would have contained high carbohydrate items like bread and pasta. Changing this is likely one of the reasons why there was a reduction in carbohydrates seen in the new menu, and although this change did not reach significance it still represents a movement towards overall improvement.

Although energy, salt, sodium, and saturated fat all exceeded their GDR in the 2022 menu, these four have significantly improved from the 2019 menu, likely due to the changes mentioned previously. Energy is now 196 kcal over the GDR, whereas previously it was 313 kcal. This improvement seen for energy was in part due to the greater variation in menu items in 2022. The 2019 menu had many food items frequently repeated across the 4-weeks. Examples include pitta bread, sandwiches, baguettes, and other carbohydrate-heavy options. While the 2022 menu has these same items, they are not repeated as frequently and do not often appear in the dinner menu options. New dinner options include recipes with higher quantities of vegetables, such as homemade pies containing vegetables and protein (e.g. fish, chicken). This reduction potentially could lead to a decrease in the risks associated with a diet high in calories, such as reducing the risk of obesity.^(42)^ Given that the prisoner populations tend to have a greater restriction for exercise opportunities, it is important to be mindful of a diet exceeding the recommendation for calorie intake. However, in this new menu the average content of calories was only slightly higher, so whether this does pose a great risk would depend on further research identifying exactly what prisoners are consuming.

Considering salt and sodium, the reduction between the two menus is significant, however, the levels of both are still quite high given the known risks associated with a diet high in salt and sodium.^(26)^ The prison population overall is dealing with the burden of being an aging population, and evidence indicates the increase in health risks associated with high salt and sodium diet for older people.^(43)^ While for young offenders the concern of aging and high salt and sodium diet may not be apparent at first, it is important to consider that there is a roughly 32.5% reoffending rate amongst young offenders as of 2021.^(44)^ The majority of those who reoffend were between the ages of 15–18, and it’s likely with continued reoffences these young offenders will move into the adult population. Regardless of whether a young offender reoffends, or stays within the general population, as earlier identified the general population does consume a high salt diet, X g above the recommendations. It would therefore be important for young offenders to consume a diet within the dietary requirements in addition to instilling healthier behaviours prior to release, to reduce the risk of high blood pressure, coronary heart disease, and osteoporosis in the long-term.^(45–47)^

Additional risks to osteoporosis include a diet low in vitamin D.^(48)^ Vitamin D is difficult to obtain through diet, and in keeping with this vitamin D was significantly below the GDR in the 2019 and 2022 menus. These results reflect previous research, for example, Mommaerts *et al.* ^(49)^ (mean = 4.84), a menu analysis by Stanikowski *et al.* ^(50)^ (mean = 5.10), and a dietary analysis by Gesch *et al.* ^(51)^ (mean = 3.50). While vitamin D was below the GDR in both menus, the 2022 menu was significantly higher than in 2019. This demonstrates the efforts made by stakeholders responsible for catering to improve the new menu. The key effort was the inclusion of a vitamin cordial juice drink, with two offered per day for prisoners, containing an additional 5ug of vitamin D. This vitamin juice drink was also a contributing factor to why zinc is now meeting the GDR in the 2022 menu. Given the status of vitamin D being difficult to obtain through diet, the use of supplements offers an alternative to achieving dietary requirements. This would be particularly beneficial in a prison environment due to prisoners’ limited access to outside spaces, which further restricts their ability to obtain vitamin D through sunlight.^(52)^

As with the improvement in vitamin D, zinc was also found to meet its GDR in the new menu, in part due to the vitamin juice drink. This now reduces the potential impacts on the immune system caused by zinc deficiency.^(53)^ Overall, the idea of supplementation in prisons is not new, and there have been many studies which have used supplements which aimed to identify improvements in aspects of mental health and behaviours.^(51,54,55)^ However, a key issue is many of these studies did not include a baseline dietary analysis, therefore whether improvements were due to participants now meeting GDRs through supplements is unclear. One study which did include blood measures at baseline, did identify that the group taking omega-3 supplements increased their blood levels of omega-3 by the end of the intervention period.^(56)^ Currently, within the author’s research group, there is further work in progress investigating the impact of vitamin D supplements in prisons, however, this work is not yet complete.

While this work has focused on assessing the menus of a YOI, the importance of food does extend beyond providing nutrition. Food is linked to social interactions and can be affected by social, cultural, and religious identities, playing a pivotal role in celebrations and bonding.^(47,57)^ During an offender’s sentence, meals provided can be a focal point of the day and provide a break from the routine and often a chance to be social with each other. With food options provided having the potential to be of varied quality, and quantity, with limited options, this can not only lead to poor physical health but have a role in mental wellbeing. Surveys of YOI offenders found that they often commented on the menu content and food quality in a negative manner. This included a lack of fresh fruit and vegetables, too many high-salt foods, processed foods which were too fatty, too many carbohydrates, and a lack of protein.^(47)^ These YOI offender comments mirroring many outcomes of this analysis.

While overall this work is able to recognise the efforts of the justice service in improving diets and a commitment towards meeting the GDRs. We do identify a few areas which need continued work to ensure that offenders are achieving optimal nutrition and health.

### Limitations and next steps

These results are specific to a single YOI, and therefore not necessarily generalisable to male or female adult prisons, or female YOIs. While similar budgets for providing food to prisoners exist across the UK, a budget of around £2 per prisoner per day, the menus are subject to the individual prisoners catering manager to devise.^(8)^ Secondly, this study consisted of a menu content analysis, therefore, these results reveal what nutrient content, on average, is available to prison residents, but cannot offer information on prisoner consumption. The collection of food diary data can be difficult in a prison setting due to issues of low literacy, as well as general limitations of food diaries such as misreporting portion sizes.^(58)^ Therefore, performing an overall menu analysis can give an indication as to the nutrition content available to prisoners.

Additionally, prisoners can purchase food items through the onsite canteen (prison shop), and these items were not factored into this menu analysis. An important next step would be to conduct a food diary analysis, within this prison, which would identify what prisoners are consuming from the 2022 menu, as well as considering any food items purchased via the canteen. Finally, a further menu analysis could be performed for male and female adult prisons.

## Conclusion

Though areas of concern were identified, the new menu has, however, made numerous attempts to offer prisoners a diet more in line with recommendations. Key areas of improvement include the provision of the vitamin juice drink which supports prisoners in meeting their recommendations for vitamin D and zinc. Additionally, the added variety in prison meals has increased fruit and vegetables and reduced total and saturated fat. Moving forward, the results of this study have led to a new breakfast pilot, where sugar will be removed from the breakfast packs, aiming to reduce total sugar content of the menu. Additionally, the results of this study have led to further efforts to improve recipes to provide prisoners with nutritious food that meets dietary guidelines.

## Abbreviations

**DRIs:** Dietary reference intakes; **DRVs:** Dietary reference values; **EFSA:** European Food Safety Authority; **GDR:** Government dietary recommendations; **HMPPS:** His Majesty’s Prison and Probation Service; **NDNS:** National Diet and Nutrition Survey; **UL:** Tolerable upper intake level; **YOI:** Young Offenders Institution.

## References

[1] Geissler C , Powers H. Human Nutrition. 13 ^th^ ed. Oxford: Oxford University Press; 2017.

[2] WHO. Nutrition. Geneva: WHO; 2022.

[3] WHO. Micronutrients. Geneva: WHO; 2022.

[4] Gómez-Pinilla F. Brain foods: the effects of nutrients on brain function. Nat Rev Neurosci. 2008;9:568–578. 18568016 10.1038/nrn2421 PMC2805706

[5] Eves A , Gesch B. Food provision and the nutritional implications of food choices made by young adult males, in a young offenders’ institution. J Hum Nutr Diet. 2003;16:167–179. 12753110 10.1046/j.1365-277x.2003.00438.x

[6] WHO. Malnutrition Fact Sheet. Geneva: WHO; 2021.

[7] Devine A , Lawlis T. Nutrition and vulnerable groups. Nutrients. 2019;11:1066. 31091644 10.3390/nu11051066 PMC6566763

[8] HM Inspectorate of Prisons. Life in Prison: Food. London (UK): HM Inspectorate of Prisons; 2016.

[9] Department of Health and Social Care. Report of the Independent Review of NHS Hospital Food. London: Department of Health and Social Care; 2020.

[10] NHS. Hospital Admitted Patient Care Activity, 2021–22. London: NHS; 2022.

[11] Gov.uk. Criminal Justice System Statistics: Sentence Types. London: Gov.uk; 2023.

[12] Wales YJBfEa. Youth Justice Statistics: 2021 to 2022: Statistics Bull. London: National statistics; 2023.

[13] Politics.co.uk. Young Offenders Institute. London: Politics.co.uk; 2021.

[14] Home Office Justice Committee. Mental Health in Prison: Fifth Report of Session 2021–22. 5 ^th^ ed. Westminister: House of Commons; 2021.

[15] Ministry of Justice. Assessing the Needs of Sentenced Children in the Youth Justice System 2018/19. Wellington: MOJ; 2020.

[16] HM Inspectorate of Probation. Mental Health. Manchester: HM Inspectorate of Probation; 2023.

[17] Commissioner Cs. Who are They? Where are They?. London: Children’s Commissioner for England; 2019.

[18] Lee P. Youth Custody: Costs: Written Question - 144303. London: UK Parliament; 2018.

[19] Public Health England. Government Dietary Recommendations. London (UK): Public Health England; 2016.

[20] EFSA. Overview on Tolerable Upper Intake Levels as derived by the Scientific Committee on Food (SCF) and the EFSA Panel on Dietetic Products, Nutrition and Allergies (NDA). Summary of Tolerable Upper Intake Levels. Parma, Italy: European Food Safety Authority (EFSA); 2018.

[21] Nutritics. Nutritics, Vol. Research Edition, v5.80. Dublin: Nutritics; 2019.

[22] Corp I. IBM SPSS Statistics for Macintosh Version 29.0 ed. Armonk, New York (NY): IBM Corp; 2022.

[23] EFSA Panel on Dietetic Products NaA. Scientific opinion on dietary reference values for magnesium. EFSA J. 2015;13:4186. 10.2903/j.efsa.2015.4186 PMC13151575 42109801

[24] EFSA. Tolerable Upper Intake Levels for Vitamins and Minerals. Parma, Italy: European Food Safety Authority (EFSA); 2006. 10.2903/j.efsa.2024.9052 PMC11538927 39507293

[25] Leyvraz M , Chatelan A , da Costa BR , et al. Sodium intake and blood pressure in children and adolescents: a systematic review and meta-analysis of experimental and observational studies. Int J Epidemiol. 2018;47:1796–1810. 29955869 10.1093/ije/dyy121

[26] NHS. Salt: The Facts. London: NHS; 2021.

[27] NHS. Fat: The Facts. London: NHS; 2023.

[28] WHO. Salt Intake. Geneva: WHO; 2023.

[29] Action on Salt. Salt and Children. Salt and Your Health. London: Action on Salt; n.d.

[30] Harvard School of Public Health. Salt and Sodium. Boston: Harvard School of Public Health; 2023.

[31] Chrisostomou C , Leach B , Njekwa B , Karfopoulou E , Tammam J. Micronutrient analysis of food provision and prisoner choice in an adult male UK prison compared to healthy eating guidelines J Hum Nutr Diet. 2019;32:15–31.

[32] Johnson C , Labbé C , Lachance A , et al. The menu served in canadian penitentiaries: a nutritional analysis. Nutrients. 2022;14:3400. 36014903 10.3390/nu14163400 PMC9416739

[33] Public Health England. Assessment of Salt Intake from Urinary Sodium in Adults (Aged 19 to 64 Years) in England, 2018 to 2019. London: Public Health England; 2020.

[34] EFSA Panel on Nutrition NFaFA, Turck D , Castenmiller J , et al. Dietary reference values for sodium. EFSA J. 2019;17:e05778. 32626425 10.2903/j.efsa.2019.5778 PMC7009309

[35] Medicine Io. Taste and Flavor Roles of Sodium in Foods: A Unique Challenge to Reducing Sodium Intake, Strategies to Reduce Sodium Intake in the United States. Washington DC: National Academies Press (US); 2010.

[36] NHS. Sugar: The Facts. London: NHS; 2023.

[37] Action on Sugar. Sugars and Obesity. London: Action on Sugar; 2023.

[38] Action on Sugar. Sugars and Type 2 Diabetes. London: Action on Sugar; 2023.

[39] Morley B , Leach B , Tammam J. Food available to people in prison from the prison shop: comparison to dietary guidelines. J Hum Nutr Diet. 2020;33:58–66.

[40] Salmon J. Dietary Reference Values: A Guide. London: HMSO; 1991.

[41] Public Health England. The Eatwell Guide. Westminster: Public Health England; 2018.

[42] EFSA Panel on Dietetic Products N, Allergies. Scientific Opinion on Dietary Reference Values for fats, including saturated fatty acids, polyunsaturated fatty acids, monounsaturated fatty acids, trans fatty acids, and cholesterol. EFSA J. 2010;8:1461.

[43] House of Commons. Ageing Prison Population: Fifth Report of Session 2019–21. Westminister: House of Commons Justice Committee; 2020.

[44] Ministry of Justice. Proven Reoffending Statistics: October to December 2021. Wellington: Ministry of Justice; 2023.

[45] Action on Salt. Salt and Osteoporosis. Salt and Your Health. London: Action on Salt; 2023.

[46] Action on Salt. Salt and the Older Population. Salt and Your Health. London: Action on Salt; 2023.

[47] HM Inspectorate of Prisons. Life in Prison: Food. London (UK): HM Inspectorate of Prisons; 2016.

[48] EFSA Panel on Dietetic Products NaA. Dietary reference values for vitamin D. EFSA J. 2016;14:e04547.

[49] Mommaerts K , Lopez NV , Camplain C , et al. Nutrition availability for those incarcerated in jail: Implications for mental health. Int J Prison Health. 2022;19:350–362. 35916664 10.1108/IJPH-02-2022-0009 PMC9757498

[50] Stanikowski P , Michalak-Majewska M , Domagała D , et al. Implementation of dietary reference intake standards in prison menus in Poland. Nutrients. 2020;12:728. 32164205 10.3390/nu12030728 PMC7146611

[51] Gesch CB , Hammond SM , Hampson SE , et al. Influence of supplementary vitamins, minerals and essential fatty acids on the antisocial behaviour of young adult prisoners. Randomised, placebo-controlled trial. Br J Psychiatry. 2002;181:22–28. 12091259 10.1192/bjp.181.1.22

[52] Gov.UK. Prison Life: Prisoner Privileges and Rights. London: Gov.UK; 2023.

[53] EFSA Panel on Dietetic Products NaA. Scientific opinion on dietary reference values for zinc. EFSA J. 2014;12:3844. 10.2903/j.efsa.2014.3844 PMC13136992 42087959

[54] Schoenthaler S , Gast D , Giltay EJ , et al. The effects of vitamin-mineral supplements on serious rule violations in correctional facilities for young adult male inmates: a randomized controlled trial. Crime Delinquency. 2021;69(4):822–840.

[55] Zaalberg A , Nijman H , Bulten E , et al. Effects of nutritional supplements on aggression, rule-breaking, and psychopathology among young adult prisoners. Aggress Behav. 2010;36:117–126. 20014286 10.1002/ab.20335

[56] Cortie CH , Byrne MK , Collier C , et al. The effect of dietary supplementation on aggressive behaviour in australian adult male prisoners: a feasibility and pilot study for a randomised, double blind placebo controlled trial. Nutrients. 2020;12:2617. 32867282 10.3390/nu12092617 PMC7551402

[57] Smoyer AB , Kjær Minke L. Food Systems in Correctional Settings: A Literature Review and Case Study. Copenhagen, Denmark: WHO Regional Office for Europe, Denmark: World Health Organization. Regional Office for Europ; 2015.

[58] Ravelli MN , Schoeller DA. Traditional self-reported dietary instruments are prone to inaccuracies and new approaches are needed. Front Nutr. 2020;7:90. 32719809 10.3389/fnut.2020.00090 PMC7350526

